# The Impact of Scientific and Technical Training on Improving Databases' Adequacy for Fetal Growth Chart Development in Limited-Resource Settings: A Case Study in the Province of South Kalimantan, Indonesia

**DOI:** 10.1155/2019/8540637

**Published:** 2019-02-03

**Authors:** Dewi Anggraini, Mali Abdollahian, Kaye Marion, Supri Nuryani, Fadly Ramadhan, Rezky Putri Rahayu, Irfan Rizki Rachman, Widya Wurianto

**Affiliations:** ^1^School of Science (Mathematical and Geospatial Sciences), College of Science, Engineering, and Health, RMIT University, GPO BOX 2476, Melbourne, VIC 3001, Australia; ^2^Study Program of Mathematics, Faculty of Mathematics and Natural Sciences, University of Lambung Mangkurat (ULM), Ahmad Yani Street, Km. 36, Banjarbaru, South Kalimantan 70714, Indonesia; ^3^Study Program of Statistics, Faculty of Mathematics and Natural Sciences, University of Lambung Mangkurat (ULM), Ahmad Yani Street, Km. 36, Banjarbaru, South Kalimantan 70714, Indonesia; ^4^Ulin Public Hospital, 43 Ahmad Yani Street, Km. 2.5, Banjarmasin, South Kalimantan 70233, Indonesia; ^5^Abdi Persada Midwifery Academy, 365 Sutoyo S. Street, Banjarmasin, South Kalimantan, 70115, Indonesia

## Abstract

**Objectives:**

To assess the impact of scientific and technical training on midwives' abilities in collecting and recording the key performance indicators for fetal growth chart development in limited-resource settings.

**Methods:**

A descriptive design was used to describe midwives' abilities in timely collecting and recording the minimum data required to estimate fetal weight and develop fetal growth chart. The study was conducted among 19 urban and rural midwives in South Kalimantan, Indonesia, between April 2016 and October 2017. The training provided access to antenatal care information on 4,946 women (retrospective cohort study) and 381 women (prospective cohort study).

**Results:**

The average amount of recorded antenatal care data on the key performance indicators of fetal growth assessment has been significantly improved (from 33.4% to 89.1%, p-value < 0.0005) through scientific and technical training.

**Conclusions:**

Scientific knowledge and technical abilities have enabled midwives to timely record routine data of the key performance indicators for fetal growth surveillance. Access to this information is vital during different stages of pregnancy. The information can be utilised as evidence-based guidelines to assess fetal risks through fetal weight estimation and to develop fetal growth chart that is currently not available in Indonesian primary healthcare systems.

## 1. Introduction

Fetal growth assessment during antenatal care (ANC) is fundamental in preventing potentially adverse pregnancy outcomes. It has been well documented that one of the main objectives of ANC services is to detect the risk of intergrowth abnormalities [1, 2]. The information can facilitate timely evidence-based interventions and ensure safe pregnancy outcomes [2–5].

Access to, adequate use of, and systematic analysis of fetal growth information is crucial for monitoring, detecting, and assessing risks linked to neonatal mortality. The access to reliable, complete, and timely information on pregnancy-related outcomes and interventions remains challenging in Indonesia. Thus, leading to non-evidence-based decisions for program planning and allocating resources [6, 7].

Access to the results of fetal growth assessment during routine ANC examinations is essential [2, 8–10]. The assessment can be carried out through fetal weight estimation [5] since there is a significant association between birth weight and fetal growth [11]. This surveillance tool can provide sufficient and effective information for early recognition of growth disturbances, informed planning, decision making, and monitoring policy progress to end preventable neonatal mortality [2, 10, 12–14].

Improvement of the data availability, consistency, and quality has become one of the world's priority actions in increasing life survival and reducing the burden of care costs for life-threatening situations and disability for newborns [13, 15–17]. Data improvement will not independently save the lives of both mother and newborn [14]. However, if the key performance indicators for fetal growth assessment are consistently monitored and recorded during pregnancy, possible complications and abnormalities can be detected and dealt with in a timely manner [13–17].

Midwives are primary practitioners (over 87%) in providing ANC service to pregnant women, detecting potential complications and abnormalities through pregnancy to delivery, and providing timely interventions and referrals [18–21]. They are also required to document the results of the visits in the local health registration systems, for example, pregnancy registers, mothers' medical cards, and maternal and child health (MCH) booklets [9]. However, it is reported that their abilities in recording the ANC examination results are low: hospital (20%) and primary healthcare (PHC) (42.5%) [8].

Access to national and local data on individual maternal, fetal, and neonatal health information during ANC remains insufficient, particularly in developing countries. In Indonesia, the difficulty in collecting the data hampered the development of both standard fetal and newborn growth charts [22]. Unrecorded, incomplete, or unavailable data on the results of ANC examination also becomes the primary cause of non-evidence-based interventions for preventable factors associated with maternal and neonatal mortality [5, 10, 16, 23].

The aim of this study was to assess the impact of scientific and technical training on midwives' abilities in documenting the minimum data required for fetal weight estimation and fetal growth chart development.

## 2. Methods

### 2.1. Research Design, Settings, and Participants

This research is the continuation of the previously published study, and the details have been provided in Anggraini et al. [24]. The primary aim was to study retrospectively and prospectively the process of ANC services in Indonesian PHC centres, particularly in documenting routine maternal, foetal, and neonatal data that are required for fetal weight estimation [25, 26] and fetal growth chart development [27–30] (Table 1). The study is a local, multicentre, and population-based project conducted between April 2016 and October 2017 in two urban and eleven rural areas of South Kalimantan which is one of the five provinces in Indonesia recording the highest neonatal mortality rate [8, 31, 32]. The project involved nineteen midwives from nineteen different urban and rural PHC centres who had a minimum of five years working experience and were recommended by the Provincial Health Department and Midwifery Association.

A descriptive design using quantitative methods was used to achieve the research aim. Midwives' abilities in collecting and recording the minimum data required for fetal weight estimation [25, 26] and fetal growth chart development [27–30] (Table 1), before and after training [24], were compared. This initiative was also meant to improve the availability, quantity, quality, and use of local data to improve antenatal risk detection [5]. The information can provide administrative and scientific guidelines for effective expansion and distribution of limited government resources in the rural areas to end preventable mortality.

### 2.2. Research Instrument and Data Collection

This study used fetal growth assessment tools which are listed in the first column of Table 1. The objective of this tabulation is to stratify maternal and fetal measurements that are routinely undertaken during ANC service. The second column represents the recommended minimum databases for each fetal growth assessment category. In the third column, unrecorded characteristics of the minimum databases recommended for fetal growth surveillance are given. In the developed electronic register, new columns to record these characteristics were created. We proposed that these characteristics should be included in the current ANC data recording and reporting systems in Indonesia. The details of research instrument and data collection procedures can be found in the previously published study [24].

### 2.3. Statistical Analyses

Descriptive statistics used to assess the performance of routine ANC data collection, particularly in documenting the key performance indicators of fetal growth assessment (Table 1), are the same as the ones explained in our previous study [24]. These include percentages of available records of the identified characteristics and a two-sample t-test to compare the performances of data documentation process. Data management and analyses were performed using Microsoft Excel 2010 and Minitab 17.

### 2.4. Ethics Approval and Consent to Participate

As a part of doctoral degree research, this study has obtained research permissions from the Indonesian national, provincial, and local governments and two ethics' clearances from the Medical Research Ethics Committee, University of Lambung Mangkurat (ULM), Indonesia (reference: 018/KEPK-FK UNLAM/EC/III/2016) and the College Human Ethics Advisory Network (CHEAN), RMIT University, Australia (reference: ASEHAPP 19-16/RM no. 19974). Information about the confidential nature of the project and a consent form (written in both Bahasa Indonesia and English) for recruitment to the study were given to the selected midwives and pregnant women (prospective study), who all agreed to participate.

## 3. Results

The results discussed below are based on information obtained from the participating midwives, who have the average age of 41 years (ranged between 29 and 56 years) and the average working experience of 19 years (ranged between 6 and 36 years). Further details on participants' characteristics are provided in Anggraini et al. [24].

### 3.1. The Impact of Scientific and Technical Training on Improving Routine Data Collection of the Key Performance Indicators of Fetal Growth Assessment

Scientific and technical training has significantly improved the average amount of recorded ANC data on the key performance indicators of fetal growth assessment suggested in Table 1 across PHC providers based on a two-sample t-test (from 33.4% to 89.1%, p-value <0.0005) (Table 2). The average amount of recorded maternal and fetal information used to estimate fetal weight and develop fetal growth charts has been improved although some of them were not statistically significant.

### 3.2. The Improvement of Databases' Adequacy for Fetal Weight Estimation

**Figure 1:**
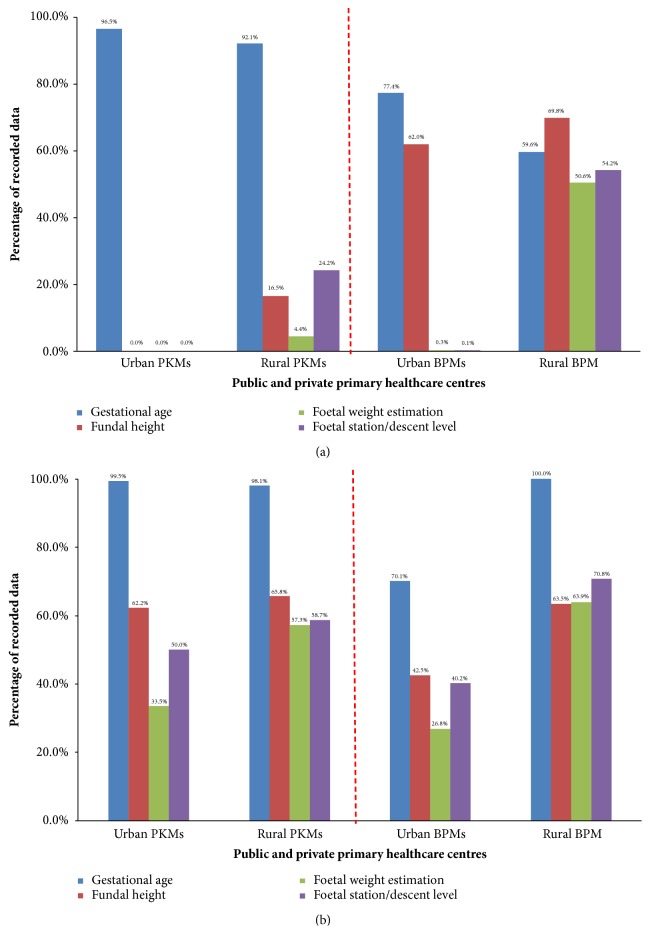
Database requirements for fetal weight estimation based on clinical method: (a) before training (retrospective data) and (b) after training (prospective data).

In Indonesia, Johnson-Toshach model [26] is nationally recognised and deployed to estimate fetal weight. This model incorporates maternal fundal height (FH) and fetal station/descent level (FS). In this paper, however, the focus is not on the accuracy of the formula that has been evaluated and discussed in the previous study [33], but on how well the information of FH and FS was recorded on the manual and electronic pregnancy registers. Figures 1(a) and 1(b), respectively, describe the availability of these characteristics before and after training.

Before training, midwives' unawareness on the importance of recording FH is clearly indicated in Figure 1(a), particularly among the public primary healthcare centers' (PKMs') midwives (0-16.5%). This was followed by low records of FS across all PHC facilities (0-54.2%). The low quality of data documentation of these important maternal and fetal characteristics hindered fetal weight estimation based on the current formula (PKMs, 0-4.4%; private primary healthcare centres (BPMs), 0.0-50.6%) precluding an intervention plan for safe delivery.

However, after training, the responsiveness to those data documentation was significantly improved (Figure 1(b)), particularly among the PKMs' midwives: FH (62.2-65.8%) and FS (50.0-58.7%). Data improvement of these characteristics promoted the improvement of midwives' competencies in estimating fetal weight and recording the results (33.5-57.3%). Although there was a slight reduction in the amount of FH records among the BPMs, this was compensated by the improved records of FS and fetal weight estimation (40.2-70.8% and 26.8-63.9%, resp.).

### 3.3. The Improvement of Databases' Adequacy for Fetal Growth Chart Development

**Figure 2:**
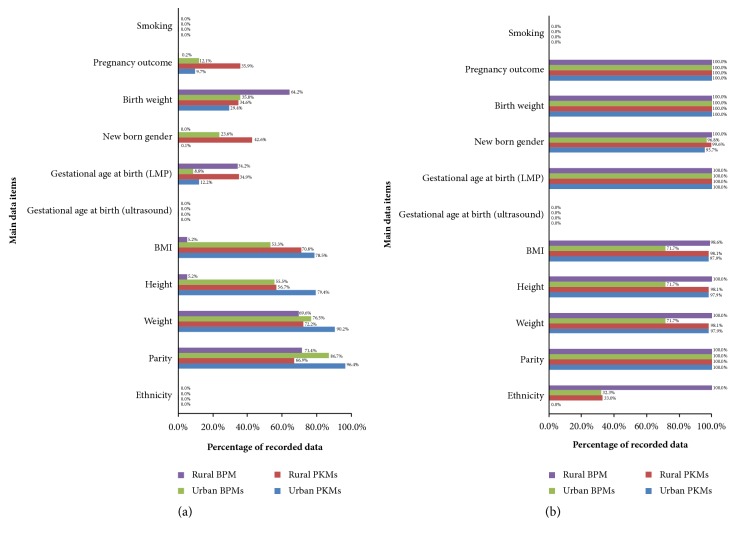
Main database requirements for constructing individual fetal growth chart: (a) before training (retrospective data) and (b) after training (prospective data).

**Figure 3:**
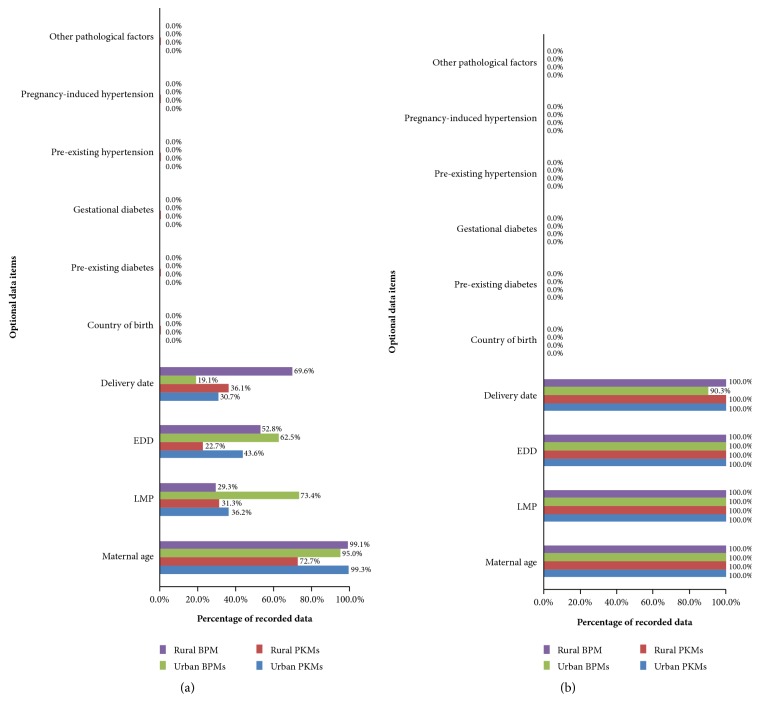
Optional database requirements for constructing individual fetal growth chart: (a) before training (retrospective data) and (b) after training (prospective data).

Gardosi et al. [30] recommended essential and optional databases to develop individual fetal growth chart based on optimal weight at a given gestational age (GA). Figures 2 and 3 present the performance of the manual (before training) and electronic (after training) pregnancy registers with respect to these recommended databases.

Figures 2(a) and 2(b) indicate that overall there was significant improvement on midwives' abilities in collecting and recording the main recommended characteristics to develop individual fetal growth chart. Specifically, information on pregnancy outcomes, birth weight, neonatal gender, and GA at birth based on last menstrual period (LMP) (from 0.0-64.2% to 95.7-100.0%) is important. This was followed by the improved records on maternal body mass index (BMI), height, weight, and parity (from 5.2-96.4% to 71.7-100%). There was no space to document ethnicity in the manual register, but it was recommended on the electronic version; thus, the records have improved from 0.0% to 32.3-100%, except for urban PKMs. There was no information on GA (based on ultrasound) and smoking habits recorded in either register.

As with the main database performance, there was significant improvement of midwives' competencies across PHC facilities in documenting the optional databases for individual fetal growth chart development (Figures 3(a) and 3(b)). This was particularly so for data access to neonatal delivery date, estimated delivery date (EDD), and LMP (from 19.1- 73.4% to 90.3-100%). There was no individual space to document information on pathological factors, pregnancy-induced hypertension, preexisting hypertension, gestational diabetes, preexisting diabetes, and country of birth in either register.

## 4. Discussion

### 4.1. The Improvement of Databases' Adequacy for Fetal Weight Estimation

The awareness by Indonesian midwives, particularly in rural areas, of the importance of documenting FH and FS at a given GA to estimate fetal weight using the current formula has significantly improved after the training (from 0-16.5% to 62.2-65.8% and from 0-24.2% to 50.0-58.7%, resp.). However, the databases' adequacy remained below 70%. A similar trend of low recording of FH (range 36-76%) was also found in rural Australia [34].

Routine and reliable measurement of FH and its documentation at a given GA throughout pregnancy are essential. Although many factors influence the wellbeing of the fetus, FH remains one of the most recommended and accessible predictors to estimate fetal weight [33] and monitor fetal growth during pregnancy [1, 35–40].

The measurement of FH is a simple and cost-effective clinical activity. It remains an important first-level screening tool, widely used during routine ANC in both high- and low-income settings, particularly in rural areas where ultrasound machines and skilled personnel are not always available [1, 41]. When the information of LMP is unreliable and ultrasonic records are not accessible, FH can potentially be used as an alternative to estimate GA, which is one of the essential measures to identify preterm birth and low birth weight (LBW) [13]. Therefore, analysis of FH records can assist midwives to improve the quality of maternal and newborn care [9, 42].

### 4.2. The Improvement of Databases' Adequacy for Fetal Growth Chart Development

Before training, access to the recommended minimum databases to develop individual fetal growth chart [30] based on local data in Indonesia was generally inadequate. These database gaps hampered the chart development for Indonesia. No such chart is currently available in the MCH booklet [43–45]. One of the objectives in this research project is to develop national fetal growth chart. However, the task requires access to larger prospective data.

The adoption of the international standard of fetal and newborn growth references [1, 46, 47] is possible and can be adjusted into individual characteristics of pregnant woman and local population [2, 5, 10]. However, nationally and geographically specific fetal growth charts are highly recommended [11, 48]. Recent studies have shown that the fetal growth chart, based on the Indonesian population rather than reference curves imported from elsewhere, can be created based on the generic global reference tool for fetal weight and birth weight percentiles developed by Mikolajczyk et al. [49, 50]. This approach was based on the notion of proportionality [28] and can be simply applied in any local population by adjusting the mean birth weight at 40 weeks of GA [50].

The current utilisation of global reference in Indonesia [50] has deployed the fetal weight estimation formula which is based on ultrasonic measurements [51]. However, ultrasonic measurements are not always accessible for our local population (Figure 2) [24], particularly in rural areas. Furthermore, a growth chart based on the ultrasonic fetal weight estimation formula should be used cautiously as maternity populations in different countries are not uniform. This may impact the optimality of fetal growth and size [10].

The provision of the fetal growth chart during pregnancy is important to assess the viability of the fetus at different stages of pregnancy and to ensure neonatal survival and wellbeing. The chart can be an effective screening tool to assist midwives in analysing and detecting the risks of fetal growth abnormalities such as prematurity and LBW, as part of their role to improve ANC service [9]. Consequently, preventive actions and referrals can be appropriately and timely initiated.

Maternal, neonatal, and child health programmes have been initiated and implemented to reduce the mortality rate in Indonesia, particularly due to prematurity and LBW [9, 52, 53]. Preterm birth, stillbirth (SB), and LBW are the major causes of neonatal mortality worldwide [8, 13, 14]. With an estimated 154 preterm births per 1,000 live births, Indonesia was ranked the 5^th^ highest for preterm births in the world [54, 55]. The occurrence of prematurity, which could be one of the causes of LBW, has increased by 3% between 1990 and 2013 and is the second most common cause of death among neonates and children under five [8, 13, 56]. However, less attention is paid on collecting antenatal information and providing surveillance tools for fetal growth during pregnancy which are crucial to improving survival and ensuring the wellbeing of newborn.

To the best of our knowledge, the scientific and technical training has, for the first time, equipped the urban and rural Indonesian midwives with the updated scientific knowledge and technology regarding fetal growth assessment during pregnancy. The training has also increased the midwives' awareness on the importance of timely documentation of the key data on characteristics of mother and fetus in the current manual pregnancy registers [24]. In relation to training, there are at least three implications. First, training can significantly improve the quality of data collected, which will help to develop accurate, Indonesia-specific charts and protocols for the surveillance of fetal growth. Next, training will be useful to implement such protocols to improve patients' safety. Finally, training will lead to better and reliable provision of local ANC data, in or near where people live, in promoting evidence-based maternal and fetal risk assessment and pregnancy outcome audit, as well as resource planning and allocation. The ultimate aim of the training is to improve the quality of healthcare services [10].

## 5. Conclusion

Scientific and technical training has significantly improved the average amount of recorded ANC data on the key performance indicators of fetal growth assessment during pregnancy. The training has equipped midwives with scientific knowledge and technical abilities to electronically record maternal, fetal, and neonatal health information during pregnancy and delivery. Provision and adequate use of this information during different stages of pregnancy enables fetal weight estimation and promotes the development of fetal growth chart that is currently not available in Indonesian ANC practices, particularly in rural areas. The fetal growth chart is an effective surveillance tool that can assist midwives in capturing, reviewing, and assessing fetal risk factors to end preventable mortality.

## Figures and Tables

**Table 1:** List of recommended minimum database requirements for fetal growth assessment.

**Fetal growth assessment tools**	**Minimum database requirements**	**Missing databases**
Fetal weight estimation	Gestational age (GA) (weeks), fetal station/descent level (FS), fundal height (FH) (cm), and fetal weight estimation (g).	None

Fetal growth chart development	Main data items:	Main data items:
	Gestational age (GA) (days not in rounded weeks) at birth based on: last menstrual period (LMP) and ultrasound (preferable), new born gender, birth weight (g), pregnancy outcome (survival status of new born), maternal weight (kg, recorded at booking/in early pregnancy), maternal height (cm), parity (recorded at booking and not including current pregnancy), ethnicity, and smoking.	Gestational age (GA) (days not in rounded weeks) at birth based on ultrasound (preferable), ethnicity, and smoking.
	Optional data items:	Optional data items:
	Last menstrual period (LMP), estimated date of delivery (EDD), delivery date, maternal age (years), country of birth, pre-existing diabetes, gestational diabetes, pre-existing hypertension, pregnancy-induced hypertension, and other recorded pathological factors (e.g. social deprivation, asthma, anaemia, substance misuse, history of small for gestational age (SGA), stillbirth (SB), and miscarriage or preterm birth).	Country of birth, pre-existing diabetes, gestational diabetes, pre-existing hypertension, pregnancy-induced hypertension, and other recorded pathological factors (e.g. social deprivation, asthma, anaemia, substance misuse, history of small for gestational age (SGA), stillbirth (SB), and miscarriage or preterm birth).

Source: [25–30].

**Table 2:** Two-sample t-test on the performance of ANC data collection on the key performance indicators for fetal growth assessment before and after midwives' training across urban and rural PHC centres.

Key performance indicators	Mean percentage of records		Mean difference (before training - after training)	t_statistics_	p-value (2-tailed)	95% Confidence interval of the difference	
	Before training	After training				Lower	Upper
***Fetal weight estimation***							

Gestational age (GA) (weeks)	81.4	91.9	-10.5	-0.9	0.379	-37.6	16.6

Fundal height (FH) (cm)	37.1	58.5	-21.4	-1.2	0.304	-73.4	30.5

Fetal weight estimation (g)	13.8	45.4	-31.6	-2.1	0.084	-68.9	5.7

Fetal station/descent level (FS)	19.6	54.9	-35.3	-2.5	0.050^*∗*^	-70.5	-0.0

***Fetal growth chart development***							

Maternal age (years)	91.5	100.0	-8. 5	-1.3	0.274	-28.7	11.7

Ethnicity/country of birth	0.0	41.3	-41.3	-2.0	0.097	-92.7	10.1

Pre-pregnancy weight (kg)	15.6	99.5	-83.9	-8.3	0.004^*∗*^	-115.9	-51.9

Pre-pregnancy height (cm)	13.9	99.5	-85.5	-11.5	0.001^*∗*^	-103.8	-67.3

Parity	80.4	100.0	-19.7	-2. 9	0.064	-41.4	2.1

Number of abortions	69.2	100.0	-30.8	-3.0	0.057	-63.3	1.8

Number of stillbirths	0.0	100.0	N/A^#^	N/A^#^	N/A^#^	N/A^#^	N/A^#^

Number of premature births	0.0	100.0	N/A^#^	N/A^#^	N/A^#^	N/A^#^	N/A^#^

The last menstrual period (LMP)	42.6	100.0	-57.5	-5.5	0.012^*∗*^	-90.5	-24.4

The estimated delivery date (EDD)	45.4	100.0	-54.6	-6.4	0.008^*∗*^	-81.6	-27.6

Gestational age (GA) at delivery (weeks)	22.5	100.0	-77. 5	-11.1	0.002^*∗*^	-99.7	-55.3

Neonatal delivery date	38.9	97.6	-58.7	-5.3	0.002^*∗*^	-85.9	-31.5

New born gender	16.6	97.7	-81.2	-7.8	0.004^*∗*^	-113.6	-48.8

Birth weight (g)	41.0	94.8	-53.8	-6.1	0.001^*∗*^	-75.1	-32.5

Pregnancy outcomes (maternal survival status)	23.2	100.0	-76.9	-8.5	0.003^*∗*^	-105.7	-48.0

Pregnancy outcomes (neonatal survival status)	14.5	100.0	-85.5	-11.3	0.001^*∗*^	-104.1	-67.0

**Overall average**	**33.4**	**89.1**	**-55.7**	**-12.8**	**0.001** ^**∗**^	**-64.3**	**-47.1**

^*∗*^The p-value < 0.05 indicates a significant difference

^#^N/A means not applicable since the standard deviations of both groups are 0.

## Data Availability

The data used to support the findings of this study are available from the corresponding author upon request.

## Abbreviations

ANC: Antenatal care
MCH: Maternal and child health
PHC: Primary healthcare
FH: Fundal height
FS: Fetal station/descent level
PKM: Public primary healthcare centre
BPM: Private primary healthcare centre
GA: Gestational age
LMP: Last menstrual period
BMI: Body mass index
EDD: Estimated date of delivery
LBW: Low birth weight
SB: Stillbirth.
